# Home Training of Girls

**Published:** 1890-01

**Authors:** 


### HOME TRAINING OF GIRLS.

Dr. Howard Crosby gives emphasis to the subject of the proper bringing-up of our girls. He says, "A primal defect in our social life is the notion that girls have nothing to do. Boys are brought up to some employment, but girls to none, except where pecuniary want compels them. The family that is 'well-off' has busy boys and idle girls. The young man, after eating his breakfast, starts out to his daily occupation, and returns at the close of the day. The young woman, after eating her breakfast, (usually at a late hour), saunters about in quest of amusements. Novels, gossip, shopping (for unnecessary trifles), dressing in three or four different costumes, formal visiting, drawing (if able), and lounging, are the elements of the young woman's day. In the evening, by way of recreation (!), she goes to the theatre or a ball.

"This unequal discipline of the sexes is the basis of innumerable evils. It makes the girl careless and selfish; it turns her mind to personal adornment and other frivolous matters as the great concerns of life; it takes away the sense of responsibility, and produces feebleness and disease in her physical constitution. It also prevents her from asserting her true dignity in the eyes of man, for the life of utility is alone dignified. Women, thus brought up in indolence, are looked upon by men very much as were the women of the old dark times of the world ; as mere playthings, expensive toys, not as counselors and friends. Marriage in such circumstances is a mere mercenary arrangement, and the girl is prepared neither in body nor mind for the serious responsibilities and lofty duties which marriage implies. Her training, moreover, or lack of training, has made it necessary for her to marry a long purse. Economy, helpfulness, co-operation—these are not coming to the new household from this vain source. Dresses, drives, entertainments—these will form the staple demands on the young husband. Accordingly in city life where this class of young woman is chiefly found, a young man is (greatly to his hurt often) kept from marrying by reason of its costliness, whereas society should be so ordered, that marriage would help the larder and not beggar it. We want simplicity of life, frugality, modesty, industry and system. If we could introduce these virtues into our higher society, we should diminish the despair, envy, jealousy, dissipation and suicides of the single, and the bickerings, wretchedness and divorces of the married.

"Let our girls have as regular daily duties as our boys. Let idleness be forbidden them. Let recreation be indeed recreation, at proper times and in proper quantities. Let us open more numerous avenues of female industry, and let every woman be clothed with the dignity of a useful life. Can such a reformation be brought about? My dear Madam, begin it yourself. Rule your household on this principle. Have the courage to defy fashion where it opposes. Be a bold leader in this reform, and you will soon see a host of followers glad to escape from the old folly."

